# The Census of 1890 and the Dentists

**Published:** 1895-09

**Authors:** William Shaw Twilley

**Affiliations:** Baltimore, Md.


### THE CENSUS OF 1890 AND THE DENTISTS.[1]

BY DR. WILLIAM SHAW TWILLEY, BALTIMORE, MD.

In August, 1890, I was approached by Doctor Richard Grady who, in a joking way, asked if I considered myself "a manufacturer," to which I replied, "No." He then gave me the first information I had received of the intention of the Census Bureau to classify dentists as manufacturers, telling me that one of the enumerators had requested and then demanded of him to reply to the questions of the manufacturer's blank. I assured him I would object to answering, and would only do so when forced, and then under protest. That week, it being the last week in August,

[1] Read before the Odontological Society of Pennsylvania, March 9, 1895.

1890, an enumerator called at my office, and when he pressed the matter I filled in the blank of name, age, occupation, and address, refusing to answer any further questions, which determination was met with a threat of prosecution in the Federal courts. I related my experience to Dr. Grady, who assured me if the profession of the city would support him he would persist in his refusal.

On the afternoon and night of September 30, 1890, I made every effort within my power to gain that support, but failed to succeed. I being the second on the list, Dr. Grady (who had in the mean time made use of the public press to call the attention of the profession to the need of early and earnest action) very kindly assured me of his support should the organizations stand by me, which in a way was done, and I am glad to say he more than fulfilled his promise, as it was by his advice and the use of the public press that the early work was put in shape and condition. Soon the Maryland organizations, with Professor R. B. Winder now as commander-in-chief, were quite actively engaged in the matter. The first official call ever issued by any organization was by the Association of Dental Surgeons of Baltimore City, September 11, 1890, calling a meeting for Friday evening, September 12, 1890.

It was there decided that, inasmuch as the Association was small in numbers, and each member thereof also a member of the Maryland State Dental Association, they would as a body resist the demands, but let the financial support come from the Maryland State Dental Association, which organization issued two calls in the month of November, 1890. At one of these meetings a resolution was adopted "that all members refuse to sign the report offered by the census enumerators, and that the sum of two hundred dollars be appropriated to defray the expenses of the dentist of whom a test case be made."

On January 14, 1891, the public press announced that a committee from one of the city organizations had visited Washington, and, after consultation with special agent Williams, had signed a paper advising the dentists of Baltimore no longer to hesitate in giving the information required. Upon their return and after legal consultation, finding that no penalty was attached for non-compliance, the public press on February 4, 1891, informed us that the same committee "repudiate their action of January 14, 1891, and withdrew their signature to the agreement." I hope soon to give the stenographer's report of this meeting.

After a seeming rest of a year, what was known as the "Willcox Bill," and styled by the press as "a bill in the nature of thumbscrews and spiked boots," passed the House of Representatives. Again the war-cry was sounded, and the Maryland State Dental Association reaffirmed its action of November 20, 1890, and appointed as a committee Drs. R. B. Winder, Sr., Edward Nelson, T. S. Waters, A. J. Volck, William A. Mills, David Genese, and W. S. Twilley, instructing them to use every honorable means possible to prevent the passage of this bill without an amendment so as to omit dentistry in all its branches from the tabulations of the census. The first official act of this committee, Professor R. B. Winder, chairman, was to urge other State dental organizations to co-operate with them for the end desired. As a result, committees from societies of New York, New Jersey, Pennsylvania, Connecticut, North Carolina, District of Columbia, and Maryland formed the "Associated Committee of the Dental Societies," Dr. John B. Rich, chairman. Many copies of the Willcox Bill were printed, and with the desired amendment attached were sent where it was thought good could be done.

I have since been told by the chief of one of the departments of the interior that they were for a time overrun with protesting letters and marked copies of this bill. From the calm following this work it was thought peace had come, but no; imagine the surprise of your humble servant one evening when, glancing over an afternoon paper, he read that the "Willcox Bill was on file in the Senate for its final reading." Hastening with the news to the ever-willing Professor Winder, he at once, with all that energy which characterized him, worked with untiring efforts for the suppression of this our common foe. The Associated Committee again commenced work, and urged each society and member thereof to bring to bear upon the individual members of the Senate all available influence. To Dr. Rich belongs, so far as I know, the credit of securing a hearing for the dentists, a task by no means to be despised or belittled. At the hearing Dr. Rich, as our spokesman, made such an oratorical and scientific effort, lauding the dentists so highly, that those manufacturers present, with the chairman, Hon. Eugene Hale, of Maine, as their leader, became somewhat aroused, and for a time it seemed as if ours was a lost cause.

Professor Winder, who had worked for two days and nights in Washington with but little sleep, appeared exhausted and unable to raise his voice in a single effort to stem the tide which was now about to carry our little craft upon the rock of destruction. "Opportunity brought forth the man," in the person of Dr. J. D. Thomas, of Philadelphia, who with great diplomacy changed the dark clouds of despair into bright rays of hope. There was yet a chance for the sinking ship, and with hope still within our hearts we sought to regain our position and succeed.

I arose, asked for five minutes of the committee's time. With a manufacturer's blank in my hand I read the questions asked and repeated the answers given. The whole thing seemed so ludicrous that Mr. Hale exclaimed, "I do not wonder you gentlemen object, if those were the questions asked."

Mr. Porter (who had travelled for two days and nights to be present at this meeting, as he afterwards remarked) replied that with the great number of men he had to employ it was impossible to avoid getting an ignorant one occasionally, and had he known of this enumerator he would at once have been discharged. To which I answered, "You could not help but know, as we were given the ordinary manufacturer's blank in printed form and you had before you our letters of objection, and yet this enumerator was kept upon the force for over eighteen months, and may be there now for all I know."

We were asked what we wanted. It was so easy to tell the "old old story,"—"To be classified with the lawyers and physicians."

Mr. Porter, realizing for the time being that he was defeated, answered, cheerfully, "Why, certainly, gentlemen, I will attend to it." Dr. Rich displayed his acuteness by demanding a "written statement to that effect right here while we are all present." Mr. Porter, who had started to leave the room, returned to his seat and wrote the original, of which this is a copy:

"The Superintendent of the Census agrees with representatives of the Dental Associations, at a meeting of the Senate Committee on the Census, to carry out the spirit and purpose of the amendment presented by the said Associations at the meeting, and which appears in the paper marked "A" hereto attached.

(Signed) "Robert P. Porter.

"June 27, 1892."

Success was now assured, victory was stamped upon our banners. Then followed hand-shaking, congratulations, and dinner with Dr. Williams Donnelly, where Drs. J. D. Thomas, of Philadelphia; Charles A. Meeker and F. C. Barlow, of New Jersey; H. B. Noble, John B. Rich, and Williams Donnelly, of Washington; R. B. Winder, William A. Mills, and William Shaw Twilley, of Baltimore, enjoyed the "feast of reason and the flow of soul."

